# An Automated, Experimenter-Free Method for the Standardised, Operant Cognitive Testing of Rats

**DOI:** 10.1371/journal.pone.0169476

**Published:** 2017-01-06

**Authors:** Marion Rivalan, Humaira Munawar, Anna Fuchs, York Winter

**Affiliations:** Department of Biology, Humboldt University of Berlin, Berlin, Germany; Radboud University Medical Centre, NETHERLANDS

## Abstract

Animal models of human pathology are essential for biomedical research. However, a recurring issue in the use of animal models is the poor reproducibility of behavioural and physiological findings within and between laboratories. The most critical factor influencing this issue remains the experimenter themselves. One solution is the use of procedures devoid of human intervention. We present a novel approach to experimenter-free testing cognitive abilities in rats, by combining undisturbed group housing with automated, standardized and individual operant testing. This experimenter-free system consisted of an automated-operant system (Bussey-Saksida rat touch screen) connected to a home cage containing group living rats via an automated animal sorter (PhenoSys). The automated animal sorter, which is based on radio-frequency identification (RFID) technology, functioned as a mechanical replacement of the experimenter. Rats learnt to regularly and individually enter the operant chamber and remained there for the duration of the experimental session only. Self-motivated rats acquired the complex touch screen task of trial-unique non-matching to location (TUNL) in half the time reported for animals that were manually placed into the operant chamber. Rat performance was similar between the two groups within our laboratory, and comparable to previously published results obtained elsewhere. This reproducibility, both within and between laboratories, confirms the validity of this approach. In addition, automation reduced daily experimental time by 80%, eliminated animal handling, and reduced equipment cost. This automated, experimenter-free setup is a promising tool of great potential for testing a large variety of functions with full automation in future studies.

## 1. Introduction

Animal models of human pathology are essential for biomedical research. These models are critical for revealing causal relationships between specific biological mechanisms and behavioural symptoms in humans. Ultimately, they are necessary for predicting drug effects or alternative therapeutics for the treatment of numerous human pathologies. A recurring issue in animal research is the poor reproducibility of behavioural and physiological findings within and between laboratories. Several studies have shown that the most critical factor involved in this issue remains the experimenter herself, who causes variability that is not eliminated by standardizing genetic backgrounds or physiological methods [1,2]. The experimenter’s influence mainly occurs through animal contact and idiosyncratic handling methods [1]. In rats, several days of repeated handling modulates the animals’ vulnerability to addiction [3], and in ovariectomized female rats it can enhance their performance in a cognitive radial-maze task [4]. The expression of anxiety-like behaviours in the elevated plus maze is attenuated in Dark Agouti rats after acute handling, but not after diazepam injections [5]. Similarly, handling mice using a container or by cupping them in the palm of the hand reduces their anxiety level compared to classical tail lifting [6,7]. In a pain study, the experimenter’s gender (male) triggered a strong physiological response, leading to analgesia in the mice [8].

**Fig 1:**
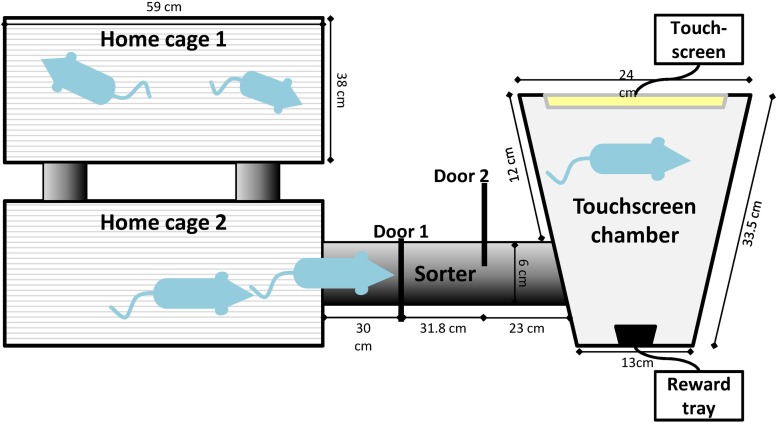
Schematic of the automated, experimenter-free operant system. The rats are permanently housed in two connected home cages. They enter the operant touchscreen chamber through an automated animal sorter (Sorter). The sorter has two guillotine gates (Gate 1 and Gate 2) and three transponder readers (R1, R2 and R3). The individual transponder chip of each rat is identified by transponder readers. The operant touchscreen chamber contains a reward tray and pellet dispenser (outside the chamber), house light, speaker and operant touchscreen. Rats can access the chamber through a 6.5 cm hole to which the sorter is connected. Not to scale; dimensions are provided.

Poor reproducibility of the cognitive abilities of rats in operant chambers has not been specifically investigated to date. However, for the assessment of rat cognitive abilities using mazes or automated-operant systems (e.g., the Morris water maze or Skinner boxes), repetitive animal handling between sessions, which brings variations in rat stress levels, could significantly affect their cognitive performance [9]. One approach to eliminating animal handling is relocating the operant testing modules to inside the home cages [10,11] of group living mice or rats. The screening of cognitive and affective abilities of group living rodents is therefore made possible in an environment in which they are undisturbed and where they follow their own cycle of activity and level of motivation for extended periods of time. A disadvantage of this approach, however, is that cage mates can influence the behaviour of others [12]. Moreover, the use of more complex operant procedures that have been standardized is not possible within a testing environment involving group access to the operant module. This is because, for a group of animals, the sequence of individual behaviours during the test cannot be subjected to a strict time schedule. Time intervals between trials (and motivation levels) cannot be controlled. In operant experimental schedules, these parameters are standardized for the assessment of discrete functions such as animal attention, impulsivity or decision-making abilities. In this study, we propose and demonstrate a novel approach using rats that combines the benefits of human-free, undisturbed group housing with that of standardized individual operant testing. Our setup consists of an automated-operant system (Bussey-Saksida rat touch system, Campden Instruments) that is connected to the home cage of group-living rats via an automated animal sorter (PhenoSys; Fig 1). The automated animal sorter functions as the mechanical replacement of the experimenter, allowing the rats to individually freely enter and leave the testing chamber using a short tunnel connected to the home cage.

Touchscreen procedures developed for rodents are well-standardized experimental paradigms, some of which directly translate to neuropsychological tests used in monkeys and humans (e.g., CANTAB). In the current study, we used the trial-unique non-matching to location task (TUNL) test. This test assesses spatial working memory in the operant chamber [13,14], and was here chosen with the later goal of integrating it into a battery of tests evaluating mild to severe cognitive impairment in aged rats. We chose the TUNL test because of its apparent complexity (TUNL acquisition phase) leading to a long acquisition phase. The underlying learning rule requiring working memory between sample and choice phase seems to be cognitively demanding for rats. Six phases of training are required before administration of the main task. Most operant procedures addressing higher cognitive functions require the ability to learn sequential, increasingly complex instrumental procedures. A demonstration of the successful, fully-automated training of rats in a complex task should encourage such an approach for all other tests developed for the touchscreen chamber.

The goal of the current study is to adapt a validated training and testing touchscreen procedure to our fully-automated home cage, sorting and operant setup. We therefore aimed to establish that self-motivated and undisturbed animals can acquire a complex cognitive task by entering the operant chamber through automated gating at a similar if not faster rate than animals manually placed. We found that this setup permits highly efficient experimentation both in terms of animal experimental time, and experimenter involvement. Such automation may be an important step toward the assessment of a large variety of functions with full automation in future studies [15,16].

## 2. Material and Methods

### 2.1 Animals

Male Wistar Han rats were used in this study (*n* = 12) and in a prior pilot study (additional *n* = 6). Rats were obtained from a commercial breeder (Harlan, Rossdorf, Germany) and were six weeks old upon arrival. Preparation for training was given according to the protocol developed by Bussey and colleagues [13]. During the first week, the rats were habituated to the lab environment (22 ± 2°C, 55% humidity), with an inverted 12–12 h dark/light cycle (lighting from 14:00 to 02:00), and were provided food and water *ad libitum*. At seven weeks of age, each rat was subcutaneously implanted with a radio-frequency identification (RFID) transponder in the groin area (under isoflurane anaesthesia). The RFID tag has a unique identification code allowing the automated individual identification of each animal using an RFID transponder reader. Two days after transponder implantation, the rats were handled and weighed daily for three consecutive days, after which they were introduced to sugar pellets (45 mg, AIN-76A, TestDiet) for two days in their home cages. The same sugar pellets were used throughout the experimental period. In addition to the experimental pellets, at 12:00 each day, six rats received 90–108 g of pellets (15–18 g per rat; chow V1535-3, Ssniff, Soest, Germany) which maintained rats at 85–90% of their free-feeding weight. Body weight was measured three times per week and growth was carefully monitored. Each experimental group in both the pilot and the main study consisted of six animals. Three groups were tested in succession; first the pilot group, then the two experimental groups. The rats were nine weeks old at the beginning of the training phase.

The novel experimental procedures described here were designed to allow for maximal animal welfare. Animals lived undisturbed as a group within their home cages. Briefly, data collection was performed using automated observational methods applied to undisturbed group-housed animals that voluntarily decided to visit the experimental chamber for the collection of rewards. The health of the animals was monitored daily. The experimental procedure did not cause any damage, pain or suffering to the animals. The animals were not sacrificed at the conclusion of the study. This study was performed under the supervision of the animal welfare officer (Tierschutzbeauftragter) at the Humboldt University. Experiments followed national regulations in accordance with the European Communities Council Directive 10/63/EU.

### 2.2. Apparatus

The home cage consisted of two regular rat cages connected by two transparent tubes and holes (Ø 6.5 cm) in the long side of the wall of each cage (Type IV, 59.5 cm x 38 cm x 20 cm; Fig 1). This dual-home cage provided a spacious cage environment for six adult rats. The standard rat’s food was simultaneously delivered into the food trays of both cages to prevent competition for food. The two connected cages could be placed onto a regular cage rack. The dual home cage was connected to an operant touchscreen chamber via a separate tube that also functioned as an automated animal sorter (ID Sorter, PhenoSys; Fig 1). The automated animal sorter consisted of a linear tube arrangement, three RFID transponder readers and two electronically-operated guillotine gates. The detection of an animal by the RFID readers and the movement of the gates were controlled by PhenoSoft software (PhenoSys). In principle, this operated similarly to an equivalent system for mice [17]. PhenoSoft recorded the time that each individual rat entered or left the sorter and the operant chamber, the start time of each session and the total duration of the animal’s stay in the operant chamber.

The operant chamber was a modified Bussey-Saksida Rat Touch System (Model 80604, Campden Instruments). The chamber was trapezoidal and composed of two black Perspex walls, an operant touchscreen facing the food magazine, a house light and a speaker situated above the chamber. A black Perspex board (mask) containing 15 windows (three rows x five columns, 3.3 × 3.3 cm) was positioned 0.9 cm in front of the touchscreen (Nexio 150A, iNEXIO CO., LTD.) in order to restrict the area available for potential animal responses during the TUNL task. The food magazine could be illuminated and included an infrared beam nose poke detector. Rats could enter and leave the operant chamber through a 6.5 cm (Ø) hole in the wall to which the sorter was connected. We used ABET II software (Campden Instruments) to control the operant task (e.g., maximum number of trials, maximum session time, inter-trial interval, delay and separation conditions).

ABET II and PhenoSoft directly communicated via a custom programmed software module. PhenoSoft ensured that only one animal entered the chamber at any one time and remained there for the entire session. ABET II assigned the correct training schedule to each individual and a session commenced when PhenoSoft signalled the entry of a rat. ABET II communicated the end of a session to PhenoSoft, which then allowed the animal to return to the home cage. The ABET II parameters included the number of trials completed, number of correct trials, number of incorrect trials, percentage of correct trials, correction trials and latencies of different types. Parameters provided by PhenoSoft included the number of sessions per training phase, the latency before entering the operant chamber and the duration of a visit to the chamber.

### 2.3 Fine-tuning of automated procedures

The goal of the pilot study was to develop the adapted conditions for training and testing, as described below. The pilot group of rats did not experience the same experimental conditions as those used later; therefore, these data were excluded from the formal analysis. Observations and experimental adjustments drawn from the pilot study are summarised in the following section.

During the pilot phase, PhenoSoft was fine-tuned to ensure that only one animal was sorted at any one time. From the home cage, a rat was identified (Reader 1) and, if the operant system was unoccupied and its individual experimental schedule permitted it, Gate 1 opened so that the animal could enter the sorter. Detection by Reader 2 and Reader 3 led to the closing of Gate 1 (Fig 1). For 30 s, both gates remained closed and, if a second animal was detected by Reader 2 or 3, the sorting sequence was aborted and Gate 1 opened to allow the rats to return to the home cage. Subsequently, if only one rat was detected in the sorter, Gate 2 opened and the rat could enter the operant chamber.

During the training phases of the pilot study, the animals could enter the operant chamber without restriction or delay between two sessions (when the operant chamber was unoccupied). During such unrestricted access to the operant chamber, we observed heterogeneous types of behaviour between animals, and even between two sessions of the same animal. An animal would, in some sessions, not interact with the screen at all or stay beyond the end of the session in the operant chamber (especially during the light phase), indicating a lack of interest in performing the task, and a propensity to enter the operant chamber for reasons other than performing the task. However, during other sessions, the rat would regularly poke the screen for rewards and reach the maximum number of trials within the session. We therefore implemented restricted access to the operant chamber to ensure that all animals had the same level of motivation to enter the chamber and obtain a maximum number of rewards during each experimental session. To accomplish this, we implemented a minimum time interval between two consecutive sessions, during which an animal was denied access to the operant chamber. This inter-session interval began when the animal exited the sorter at the end of a session. Furthermore, to maintain spontaneous explorative behaviour and sustain animal motivation to enter the operant chamber, we limited the number of trials per session. During the first training phase with the activated sorter (Training 2, see below), we combined a short session duration (10 min or 17 trials) with a short inter-session interval (30 min) to ensure frequent entries to the operant chamber and to provide further opportunities for training. Training 2 is a critical learning phase. However, once the animals had learned the association between the instrumental response and the delivery of the reward, the inter-session interval during the phases from Training 3 onwards (see below) increased. For the automated procedure, the two parameters of inter-session interval and maximum number of trials per session proved to be important for homogenising the behaviours of the animals when in the operant chamber, and to motivate regular visits to the chamber.

We also modified the inside of the operant chamber after considering the conditions in Talpos et al. (2010). A small moveable shelf was originally mounted in front of the screen. This shelf had been used in other studies with the aim of helping the animal focus on the task by supporting only their forepaws and thereby preventing impulsive responding. After removal of the shelf during the pilot study, rats that had not previously poked the screen began spending more time closer to the touchscreen and eventually began to poke it. The mask used throughout our study was a standard rat mask (3 x 5 cm) supplied by Campden Instruments, and was identical to those used by Oomen et al. (2013; S1B Table). This differed from Talpos et al. (2010), who altered window size between the training and test phases (S1B Table).

### 2.4 Training and Testing

Training and testing followed established TUNL touchscreen protocols [13,14] with adjustments for our automated system (S1A and S1B Table). During a training session, the house light was off. However, upon delivery of a reward pellet, the food tray was illuminated and a brief high-pitched tone sounded (0.5 s). At the conclusion of a session, all windows on the screen turned off, and the magazine light remained off. No other stimulus signalled the end of the session. Once a rat had fulfilled the acquisition criterion (unless more than half the group still required sessions to reach the criterion), the rat could immediately begin the following training phase. If at least half of the animals were still required to complete sessions in order to fulfil the criterion, access to the operant cage was permitted to the late-learners only for a certain period (e.g., the following 4 h). This procedure aided the rats in reaching the level of the remaining group members by increasing their chances to access the operant chamber and to perform the remaining sessions to meet the criterion. Sessions were voluntarily commenced by the animals during dark or light phases.

#### 2.4.1 Training 1: Habituation/exploration phase

The goal of this phase was to habituate the rats to the as-yet-unfamiliar operant chamber and sorter and for them to associate the operant chamber with the presence of appetitive food rewards. The sorter was connected between the home cage and the operant chamber; however, all gates were open for 24 h. In this “open-sorter” condition, multiple animals could simultaneously enter the operant chamber. Before both gates were opened, three to five sugar pellets were manually placed at both ends of the sorter to motivate a first visit to the sorter and the operant chamber by an animal. In contrast to classical touchscreen training procedures [13], the animal did not experience the operant chamber in an inactive state (i.e., without screen stimuli but free pellets in the food tray). In the current study, we used the presence of multiple animals in the chamber from the commencement of the study to habituate the rats to the operant chamber and to potentially facilitate the acquisition of the instrumental response (i.e., screen poking and pellet feeding after delivery) of the animals by means of observational learning [18]. Thus, in the operant chamber, all 15 touchscreen windows were lit. Poking a lit window resulted in the delivery of two reward pellets; the windows then turned black. If no nose poke was made within 15 min, all windows turned black and one pellet was delivered. After pellet collection and a subsequent 30 s inter-trial interval (ITI), a new trial began (all windows were again lit). The parameters measured for each animal in this phase included the latency of entering the operant chamber for the first time since the commencement of training, the number of visits to the operant chamber, and the mean duration of a visit (S2 Table). A visit to the operant chamber was defined as the detection of the rat at Reader 2, followed by two subsequent detections at Reader 3. This sequence occurred when an animal entered the sorter from the home cage, travelled to the operant chamber and subsequently exited it. The duration of stay in the operant chamber was calculated as the time between the first and second detections at Reader 3. The total number of trials was recorded for the group of animals.

#### 2.4.2 Training 2: Initial touch

The goal of this phase was to habituate rats to gate movement and to allow the rats to individually learn to associate a screen touch with a larger reward. During this and the following phases, the sorter was activated so that only one rat could enter the operant chamber at any one time (section 2.2, S1A Table). In the operant chamber, all 15 windows were lit and poking a window resulted in all windows turning off and the delivery of two pellets (active trial). If no window was poked within 30 s, all windows turned off and one reward pellet was delivered (passive trial). An ITI of 20 s began after pellet collection, and a session ended after either 10 min or 17 trials. During a 30 min minimum intersession interval, an animal was not granted re-entry to the operant chamber. The parameters measured are listed in S2 Table. The percentage of active trials per session was calculated as the total number of trials in which a nose poke was made on any lit window divided by the total number of trials in this session. Prior to commencing the following training phase, animals must meet the acquisition criterion of 17 trials per session for two sessions.

#### 2.4.3 Training 3: Touch all lit windows

The goal of this phase was for the animal to associate a nose poke on a lit window with a reward. In the operant chamber at the beginning of a trial, 15 windows were lit (S1A Table). Upon one poke, the screen turned off and one reward pellet was delivered (trial). In contrast to the previous training phase, no reward was delivered if no poke was made on the screen, and the lit windows remained on until a response was made. After pellet collection, an ITI of 20 s began. A session ended after either 30 min or 50 trials. The minimum intersession interval was 2.5 h in order to reduce satiety, increase motivation and theoretically allow all animals to complete one session in the meantime (30 min per session x 5 remaining rats = 2.5 h). The parameters measured for each animal were identical to those in Training 2 except for the percentage of correct trials per session (not the percentage of active trials, S2 Table). This was calculated as the total number of trials initiated by a nose poke to any lit window divided by the maximum number of (50) trials in this session. Prior to entering the following training phase, animals were required to meet the acquisition criterion of 50 trials per session in two sessions within 24 h.

#### 2.4.4 Training 4: Touching one lit window

The goal of this phase was for an animal to learn to poke the single, lit window and to force it to respond to different windows on the screen, avoiding the potential development of a spatial bias. Each session commenced with the presentation of a randomly chosen lit window (S1A Table). Upon a nose poke to this window, the screen turned off and one reward pellet was delivered (trial). A lit window remained on until a response was registered. An ITI of 20 s began after pellet collection. Pokes to other windows were recorded, but did not end the trial. A session ended after either 30 min or 50 trials. The minimum intersession interval was 2.5 h. The parameters measured for each animal were identical to those in Training 3 (S2 Table). The acquisition criterion was 50 trials per session in two consecutive sessions.

#### 2.4.5 Training 5: Initiating

The goal of this phase was for the animals to learn to initiate a trial by first poking the food magazine. At the commencement of a session, the food magazine was lit and contained a pellet. Poking the magazine for the pellet resulted in a clicking sound and the illumination of one window (random position). Identical conditions to those in Training 4 then applied. After the ITI, the food magazine was again illuminated and the rat was again required to poke it which started the next trial with a clicking sound and one illuminated window. Pokes to an unlit window were recorded but were inconsequential. A session concluded after either 30 min or 50 trials, and the minimum intersession interval was 2.5 h. The parameters measured were identical to those in Training 4 (S2 Table), plus the number of incorrect nose pokes per session (to unlit windows). The acquisition criterion to enter the following training phase was to complete 50 trials per session within two sessions.

#### 2.4.6 Training 6: Punishment for incorrect choices

In order to reduce the number of incorrect choices, all parameters were set as in Training 5. However, incorrect responses (nose poke to an unlit window) ended the trial, which was followed by a low-pitched tone (0.5 s) and a timeout of 5.0 s with the house light turned on (incorrect trial). After an ITI of 20 s, the following trial began with a new location for the lit window (no correction trials). A session ended after either 30 min or 50 trials, and the minimum intersession interval was 2.5 h. The parameters measured for each animal were identical to those in Training 5, except for the percentage of correct trials per session. This was calculated as the total number of correct trials (nose poke to a lit window only) divided by the total number of trials per session (correct and incorrect). The acquisition criterion to enter the following training phase was to complete 50 trials with 80% of choices being correct in two consecutive sessions.

#### 2.4.7 Trial-unique non-matching to location task (TUNL) acquisition

The goal of this test was to measure the ability of a rat to remember the location of a stimulus displayed during the sample phase and to avoid the same location during the subsequent test choice phase. At the commencement of a session, the reward tray was lit and contained a pellet. Poking the tray extinguished the light, triggered a clicking sound and initiated the sample phase. Only one window was lit, and upon poking the window, the stimulus was turned off and a delay of 2.0 s began. After the delay, the food tray was lit and, in one third of cases, a single reward pellet was delivered. A poke into the tray extinguished the tray light and initiated the choice phase. Two windows were lit; the now incorrect previous sample location (S-) and the reinforced novel choice location (S+) were pseudo-randomly assigned. Poking a black window had no consequences; poking the S+ was rewarded and poking the S- was followed by a low tone (0.5 s) and a timeout of 5.0 s with the house light on. An ITI of 20 s began after reward collection or after the timeout and was followed by the illumination of the reward tray. The following trial began after poking the lit reward tray. Incorrect decisions were followed by a correction trial in which the combination of stimuli from the previous choice phase was again presented. Correction trials were repeated until the correct choice was made. In the choice phase, the lit windows were separated by 0, 1, 2 or 3 windows, displayed on any of the three rows. Distances were pseudo-randomly selected so that each distance between 0 and 3 was selected at least twice during a block of 20 trials. The duration of a TUNL session was 60 min or 84 trials. During the TUNL task, rats were allowed into the operant chamber twice within 24 h with a minimum individual intersession interval of 7 h to reduce satiety, increase motivation and theoretically allow all the animals to complete one session in the meantime (1 h per session x 5 remaining other rats = 5 h + 2 h margin). The parameters measured for each animal included the number of sessions, the number of trials per session, the duration of each session, the number of correct nose pokes per session, the number of incorrect pokes, the number of correction trials, and the time spent in the chamber in total and after task completion. The latency period before making a correct choice was the interval between exiting the magazine after initiating the choice phase and making a correct response on the screen. The latency period before reward collection was the interval between making the correct response and poking into the food tray. Animals were given a minimum of 20 sessions in order to be able to compare the progression of the animal’s performance to the data published by Talpos et al. (2010). The acquisition criterion was met when rats had reached the level of 70% correct responses (all trials) in two consecutive sessions.

#### 2.4.8 TUNL probe session

The goal of the probe sessions was to evaluate the effect of an increased delay from 2 to 6 s in rat performance between the sample and choice phases at the different S+ and S- distances. The conditions were similar to the TUNL acquisition conditions (session duration was 60 min or 84 trials, with an ITI of 20 s). Rats were allowed into the operant chamber twice within 24 h, with a minimum intersession interval of 7 h. Zero to three windows separated the two active locations (S+ and S-) and each of the three spatial separation distances, “small” (0), “medium” (1 or 2) and “large” (3) were tested with both 2 and 6 s delay conditions. The six probe test conditions were presented during separate sessions and each was repeated once, resulting in a total of 12 sessions for a single animal. Within a session, the delay and stimulus distance remained constant. We also performed an interference test to evaluate whether the stimulus location of the previous trial influenced the performance of the current trial. For this test, the ITI was reduced from 20 s to 5 s to increase the likelihood that the previous trial transferred its influence to the current trial. The delay between the sample and choice phases was 2 s, and spatial separation between stimuli was large (allowing easy discrimination). The parameters measured for each animal were identical to those in TUNL acquisition. The percentage of correct trials at small, medium and large separations was measured, as well as the latency in making a choice during the choice phase and the latency in collecting the reward.

### 2.5 Data analysis

Statistical tests were performed using R [19] and open access macros for non-parametric tests (Anastat.fr). We chose to perform nonparametric tests as the sample size of the experimental groups (*n =* 6, or *n =* 12 when the two groups of six rats were pooled) was too small to confidently assume that each dataset was normally distributed. We first tested whether the results obtained for our two experimental groups (each *n =* 6) differed to determine whether the data could be pooled for subsequent analyses. For each parameter measured in each training and testing phase, we compared the results between the two experimental groups using the Mann-Whitney test for independent samples (U) and then applied Bonferroni correction to control for the effects of multiple testing (S3 Table).

As the two experimental groups did not differ, the results for the 12 animals were pooled for the main analyses. The non-parametric Friedman test for repeated measures was performed to test for changes in dependent measures across conditions or blocks of sessions. The pairwise comparison between blocks (or conditions) was completed using the Wilcoxon signed-rank test. The results are illustrated as boxplots; to complement the description of our results the values given in the Results section are provided as mean ± standard deviation. As a rule, no statistical analysis was performed when the position of the median and of the first and third quartiles of the boxplots overlapped, as this indicated similar distributions of the datasets (as an example, see Fig 5B). Data were excluded from time periods in which technical problems were experienced.

## 3. Results

Training phases 1 to 6 were completed within seven to nine days. The TUNL acquisition task was then completed within an additional 10 to 11 days for both groups of six naïve rats using a single touchscreen system. In most cases, all rats from a group reached the criterion of a training phase on the same day. Session performance was independent of the time of day.

### 3.1 Repeatability of experimental results between groups

The two groups of six rats were then tested to determine whether there was a group effect on the results from the training phases and the TUNL task (Mann-Whitney test and Bonferroni correction for multiple testing). After Bonferroni correction, no difference was observed between the two experimental groups (S3 Table); therefore, we pooled the data from both experimental groups for subsequent analyses.

### 3.2 Rapid habituation to operant cage and sorter

**Fig 2:**
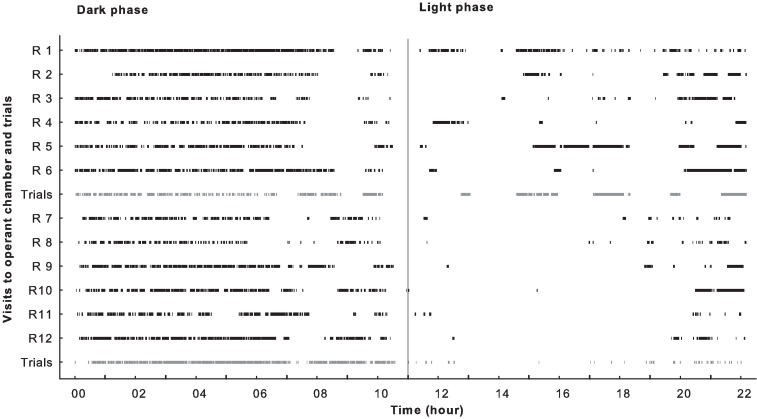
The number of visits to the operant chamber and trials made by each rat during the initial 24 h of the habituation/exploration phase. Each vertical black line indicates a visit to the operant chamber by an individual rat (R). Grey lines (upper: rats 1–6, lower: rats 7–12) signify completed trials and delivered rewards. Rats 1–6 and 7–12 were in separate groups. The vertical black line at 11 h indicates the shift from the dark to light phase. On the x-axis, “00” represents the beginning of the training phase for all rats (all introduced simultaneously to the system). The time of day corresponding to experimental time “00” was 15:00 h.

During Training 1, all rats entered the unfamiliar operant chamber within the first 15 min (4 min 46 s ± 4 min 59 s; *n =* 11 rats; one rat entered after 73 min) by passing from the home cage through the sorter tubes. After one rat had entered the sorter and operant chamber, all other rats quickly followed. At the commencement of this phase, more than two rats could simultaneously be seen in the tube. Individuals made 211 ± 28 visits to the operant chamber during the first 24 h, with 88 ± 3.7% of these occurring during the dark phase (Fig 2). A visit during the dark phase lasted 62 ± 15 s, which was on average four times shorter than during the light phase (262 ± 208 s). Almost all pellets (98%) delivered during the dark phase were triggered by touching a lit window on the touchscreen.

In Training 2, the sorter was active and each rat was mechanically sorted (gates moved regularly during the sorting procedure, as described in section 2.2) to enter the operant chamber and start a session. Animals required an average of four attempts (4.10 ± 1.45; *n =* 10) to enter the sorter until they were correctly sorted; they then proceeded to their first individual training session (two rats required more than four attempts). Once the sorting procedure concluded and an animal arrived at the operant end of the sorter, the first session commenced within 41 ± 10 s.

### 3.3 Automated touchscreen training and exiting the operant cage

**Fig 3:**
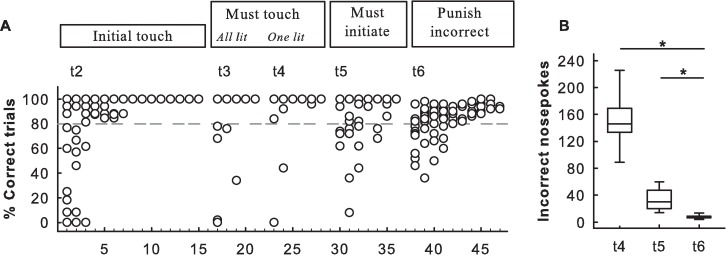
Automated touchscreen training. (A) Percentage of individual correct trials across Training phases 2 (t2) to 6 (t6). White circles show individual performance during a session. Circles can overlap. In t2, the percentage of correct trials is also referred to as the percentage of active trials (see Methods). Difficulty increases from t4 to t5 when rats learn to self-initiate a trial by poking into the pellet magazine. In t6, incorrect trials are punished with a time out. Data are based on *n* = 12 individuals. Due to technical problems, six rats continued with t2 for a further eight sessions although they already had achieved > 80% correct trials (grey dotted line; *n =* 12 sessions 1–5 and *n =* 6 sessions 8–15). (B) The number of incorrect nose pokes (nose pokes to unlit windows) during the final three phases of training. Boxplots show median, quartiles and 10^th^/90^th^ percentiles. *p < 0.05 between t4 and t6, and t5 and t6

All rats quickly reached a high level of performance in each training phase. The rats achieved more than 80% correct trials after four sessions in Training 2 (in three days), three sessions in Training 3 (in one day), three sessions in Training 4 (in one day), four sessions in Training 5 (in one to two days) and five sessions in Training 6 (in one to two days; Fig 3A). In Training 5, a poke into the food tray was required to initiate the trial. In Training 6, incorrect responses were punished. Incorrect touches to the screen significantly decreased from Training 4–6 and Training 5–6 (Fig 3B; Friedman test on the repeated (averaged) data of each training, Q(2) = 9.21, p < 0.01 and pairwise comparison using Wilcoxon signed-rank test, between t4 and t6, and t5 and t6, p < 0.05). Importantly, the rats did not spend unnecessary time in the operant chamber after the conclusion of a session. The mean latency time prior to exiting the operant chamber after the conclusion of a training session (all trainings included) was 2.6 ± 3.1 min.

### 3.4 Influence of automation on performance in the TUNL task

**Fig 4:**
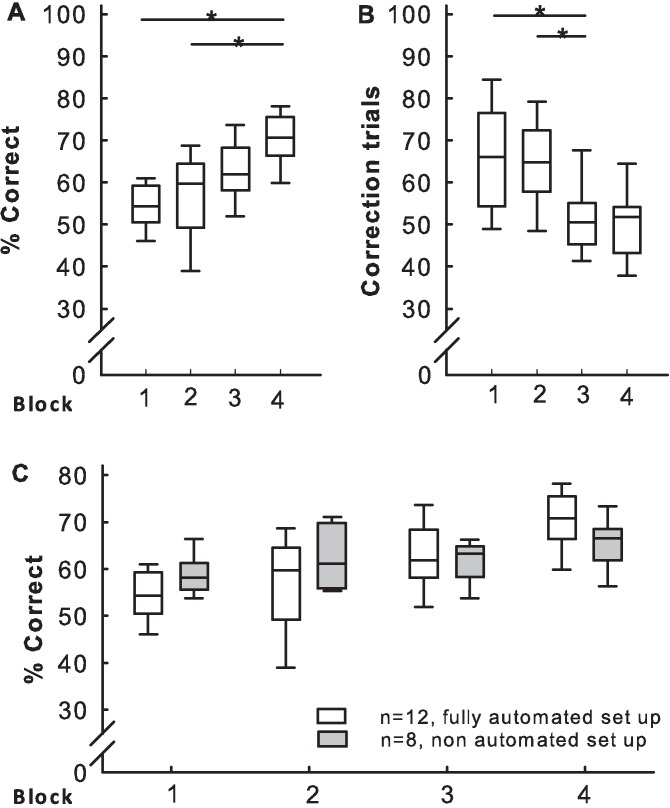
Automated TUNL task. (A) Percentage of correct trials and (B) number of correction trials averaged for blocks of five sessions. Data from *n* = 12 individuals. (C) Comparison of performance (correct trials across blocks in the TUNL task) between this study (white symbols; identical data as in A) and the published data from a study by other authors using a non-automated setup (Talpos et al., 2010; grey symbols). Boxplots show median, quartiles, and 10^th^/90^th^ percentiles. *p < 0.05

During the TUNL task, the rats achieved the learning criterion of 70% correct responses within a session, in 10–19 sessions (median = 16) and 5–10 days (median = 8). During the TUNL task, the number of correct trials (%) increased with experience (Friedman test, Q(3) = 11.34, p < 0.01 and pairwise comparison using Wilcoxon signed-rank test between blocks 1 and 4, and 2 and 4, p < 0.05; *n =* 12 rats; Fig 4A), while the number of correction trials decreased with experience (Friedman test, Q(3) = 11.34, p < 0.01 and pairwise comparison using Wilcoxon signed-rank test between blocks 1 and 3, and 2 and 3, p < 0.05; Fig 4B). These data were compared with data published by Talpos et al. (2010), who used a non-automated procedure with rats manually introduced into operant chambers for daily sessions (Fig 4C). During task acquisition, there was no difference in the level of performance between our study and the control group of Talpos et al. (2010; Mann-Whitney tests). However, the 20 sessions were completed within 10–11 days (10.8 ± 0.4 days) in our system, which was half of the days necessary to learn the task in the group of Talpos et al. (2010; one session per day and 20 days for all animals). Interestingly, rats reached 70% correct responses within 5.0 ± 3.2 days, which is similar to the 5.0 ± 3.5 days in Talpos et al. (2010). In our study, the mean latencies before making a choice (5.0 ± 2.5 s) and collecting the reward (2 ± 12 s) were very short (*n =* 6; for 6 other animals these parameters were incorrectly recorded). A trial was completed within an average of 47 ± 7.0 s, and this duration was stable across all sessions of the TUNL acquisition task.

### 3.5 Performance during the probe and interference tests

**Fig 5:**
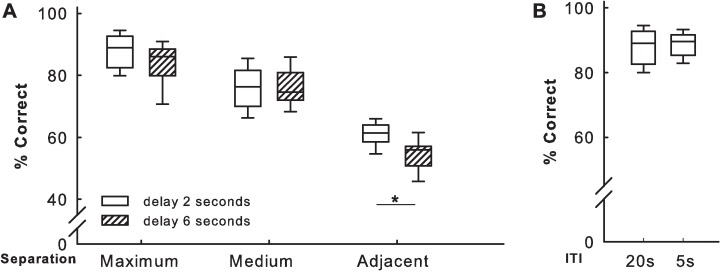
Performance during the automated TUNL probe test. (A) Performance with two different time delays (2 or 6 s) and three spatial separation conditions (maximum, medium or adjacent). (B) Performance during maximum spatial separation and a 2 s delay with different ITIs (interference test). Boxplots show median, quartiles, and 10^th^/90^th^ percentiles. *n =* 12 rats per condition. *p < 0.05

In the TUNL task, the ability to cope with various levels of difficulty was evaluated in probe sessions in which two parameters varied: the spatial distance between the S+ and S- locations from maximum (easy) to adjacent (difficult to discriminate), and the delay between sample and choice phases (2–6 s), which increased working memory demand. Performance decreased as discrimination of the two stimulus locations increased in difficulty (separation distance from maximum to adjacent) for both delays (Friedman test, Q(2) = 13.82, p < 0.001; Fig 5A). Performance was lowest for the adjacent condition, and in this condition, rats performed better with the shorter, 2 s, delay (Wilcoxon test for paired samples with Bonferroni correction for multiple testing w(12, 0.025) = 14, p’ < 0.05; Fig 5A). In the interference test, which tested for a potential interference effect between trials, the shorter ITI of 5 s instead of 20 s did not affect the level of performance (Fig 5B). The motivation to collect reinforcement measured as latency in reaching the food tray after a correct choice did not differ between probe test conditions. These latencies were on average 1.0 ± 0.64 s. The latency before making a correct choice in the choice phase was also unaffected by the different delay and stimulus distance separation levels (an average of 5.0 ± 2.4 s).

**Fig 6:**
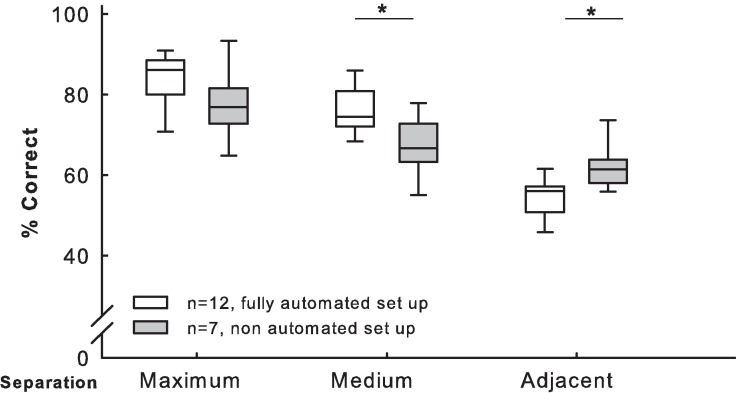
Comparison of automated versus non-automated procedures of the TUNL probe test. Both studies used a delay of 6 s. Data were obtained using an automated (current study: *n* = 12, striped boxes) versus a non-automated (Talpos et al., 2010: *n* = 7, grey boxes) system. The number of windows differed between studies (current study: three rows of five; Talpos et al., 2010: two rows of seven). The classification of the distance separations by the number of stimuli separating the lit windows during the sample and the choice phases of the test also differed between the studies (current study, Adj (0), Med (1, 2), Max (3) and Talpos et al., 2010, Adj (< 3), Med (3 ≥ n < 6), Max (≥ 6)). Boxplots show median, quartiles, and 10^th^/90^th^ percentiles. *p < 0.05

In the probe test, the performance of rats in the current study differed from the results of Talpos et al. (2010; both studies using a 6 s delay condition) regarding the two more challenging conditions of separation (medium and adjacent; Mann-Whitney with Bonferroni correction for multiple testing U = 15, p’ < 0.05 and U = 16, p’ < 0.05, respectively; Fig 6). In fact, the performance of our experimental rats was higher during the medium condition compared to the results in Talpos et al. (2010). However, in the most difficult condition, the performance of our rats was lower than that previously observed.

## 4. Discussion

In this study, we developed and validated the use of an automated, experimenter-free system for the standardized operant cognitive testing of rats. Rats were given the freedom to choose whether and when to enter the operant chamber directly from their home cage, and they did so regularly to perform the tasks at a stable level of performance. They then promptly left the operant chamber after the conclusion of a session. All rats achieved a high level of performance within a few days in each training phase and in the TUNL task. In most cases, all rats reached the criterion of a training phase on the same day. This automated, experimenter-free system yielded comparable results both within (performances were similar in both experimental groups of the current study) and between (performance in the current study and the previously published study of Talpos et al., 2010) laboratories, indicating great potential for testing a large variety of functions with full automation in future studies.

### 4.1 Specificities of the automated procedure and data collection

The experimental conditions on the first training day (unrestricted access to the operant chamber for 24 h) proved efficient at overcoming any hesitation by the animals to enter the sorter and operant cage. Taking advantage of the rats’ natural tendency to follow each other, all animals entered the system as soon as they were given access to it, interacted with the touchscreen and retrieved pellets from the food tray (Fig 2). Familiarisation of the animals with the system was most likely facilitated by the possibility of making high frequency visits of short duration to the operant chamber. A further indicator of the low level of stress toward the automated system and the sorter was in Training 2, where a short latency period prior to entering the sorter was observed the first time it was activated, and a negligible time lapse was seen before entering the operant chamber from there. This was confirmed in all subsequent training phases, with the persistence of regular, voluntary individual visits to the operant chamber.

Frequent visits to the operant chamber during training were also sustained by the combination of short training sessions and intersession intervals (section 2.3). Under these automated conditions, the animals rapidly learned the requirements of each training phase (Fig 3). In Training 5, a poke into the food tray was required to initiate the trial. During Training 6, incorrect pokes were punished. These additional steps slightly complicated the instrumental sequence of actions and may explain the higher number of sessions that were required to meet the criteria in Training 5 and 6 (Fig 3A). All rats achieved the acquisition criterion of each training phase in one or two days (three days in Training 2). This was remarkably fast considering that all rats in a group (each *n =* 6) only had access to one operant chamber.

In addition to their strong motivation to enter the system, several parameters (e.g., short latency prior to making a choice and collecting a reward, short duration of a trial and short exit latency), proves that the self-motivated start of a cognitive task did not affect the animal’s efficiency during the task. It also indicates that the motivation between animals and sessions was stable. These points are important in establishing the validity of this automated and self-initiated, experimenter-free method. The rats mostly used the dark phase (as opposed to the light phase) of the day, which can be expected for a nocturnal animal (Fig 2).

### 4.2 Adaptations of the TUNL training procedure

Training 2 (initial touch) of the TUNL training procedure differed from Training 3 (must touch, all lit) only in that a pellet was given after 30 s of inactivity in Training 2. The animals had already learnt to touch a window at the end of Training 2 and were no longer using the “free” pellet option (Fig 3). Thus, they were already fully conforming to Training 3 requirements at the end of Training 2. In such a situation, one might consider keeping phase 3 of TUNL training very brief or omitting it. In Training 6, as described in Oomen et al. (2013), punishing incorrect choices with timeouts should have increased efficiency during the TUNL acquisition phase. We found no difference in performance levels at the beginning (first block) of the TUNL test between our current study (which included a training stage to punish incorrect choices) and the TUNL data of Talpos et al. (2010), which did not include such a training phase (Fig 4; S1A Table). However, this lack of facilitation effect on the TUNL performance (first block) could also be due to method differences between our and Talpos et al. (2010) study. It might then be considered to keep Training 6 (punish incorrect) very brief or even omit it from our automated setup. However, one should keep in mind that the existing protocols developed over the course of 20 years consist of different phases based on fundamental psychological processes. The important experience an animal makes during Training 6 is to learn about the consequence of an ‘incorrect response’. It should be avoided that an animal makes such an experience for the first time during the actual cognitive testing since this would introduce a confounding factor.

### 4.3 Comparison of results within and between laboratories, and advantages of the automated and standardized approaches

Rat performance during training and testing phases in our fully-automated system was comparable between the two experimental groups (S3 Table), and was also comparable to results obtained in a less-automated system (Figs 4 and 6) [14].

The level of performance observed here in the TUNL task acquisition (Fig 4) and TUNL probe tests (Figs 5 and 6) was as expected. However, performance was higher when choice and sample stimuli were further apart, both with shorter and longer delays between sample and choice phases (Fig 5A). The interference test, which evaluates the influence of the previous trial on the performance of the current trial (Fig 5B), showed that the position of the previously correct window did not affect the following choice, in agreement with previous finding [14,20]. While the majority of training sessions in previous studies had a duration of 60 min (S1A Table), most of our sessions had a duration of 30 min, and only 10 min for Training 2. Under manual training conditions, an animal usually had only one training session per day, requiring 10 to 30 days to complete the training [13]. In contrast, in our automated system, only seven to nine days were required to complete a comparable amount of training. Likewise, TUNL training required 20 [14] to 30 or 35 [13] days with manual transfer of the rat; only 10 to 11 days was required in our automated system. In comparison with previously published data [14,20], our results confirm the validity of this fully-automated approach for rapid and efficient cognitive testing of undisturbed groups of rats. TUNL task acquisition was achieved in less days than reported in previous studies [14], and training the animals took a little longer than a week, which is a very short length of time for this type of instrumental learning procedure.

Interestingly, while the albino strains of rats are commonly considered to perform less efficiently in cognitive tasks than pigmented strains, the albino Wistar Han rats (WH) used in this study performed at the same level as the pigmented strain used by Talpos et al. (2010; Lister Hooded, LH; Fig 4C). WH rats perform worse than LH rats when detection of visual detail is required, due to their poor visual acuity [21]; however, WH rats equal LH rats in performance in a spatial discrimination task [21]. Further studies are required to determine whether the good WH rat performance is due to the shorter delay used in our study (2 s) compared to that used in Talpos et al. (2010; 6 s) during the TUNL acquisition task, procedural differences (distance between stimuli, total number of windows) or a facilitating effect of our automated conditions. The rats acquired the TUNL task in approximately half the number of days reported in Talpos et al. (2010), with a level of performance comparable between both studies for blocks of five sessions (Fig 4C). However, our rats reached the level of 70% correct choices after an average of five days (approximately 10 sessions), while this level was reached after approximately five sessions in Talpos et al. (2010). These observations could explain the lower median of our results in blocks 1 and 2 (Fig 4C) compared to Talpos et al. (2010). Procedural differences (distance between stimuli, total number of windows) may be the reason for these variations.

Surprisingly, at the medium condition of separation in the TUNL probe test, the performance of the rats was higher in our study than that reported in Talpos et al. (2010; same delay for both experiments), but declined more dramatically when conditions increased in difficulty (Fig 6). Thus, our WH rats may have been more sensitive to the increasing difficulty of test conditions. As our rats reached a somewhat higher level of performance than the pigmented rats in the easier conditions [14], our results exclude the possibility that WH rats consistently perform inferiorly to LH rats. Such an increase in variability between the groups under the more challenging test conditions is not uncommon. It remains to be seen whether there is a true variation in task performance between rat strains.

The development of procedures that do not require human handling is a promising avenue for increasing standardization and repeatability between studies and, thus, in increasing the translational validity of potential therapeutic effects [22,23]. Our automated, experimenter-free approach is therefore a promising tool for drug testing studies that usually require multiple and lengthy testing phases.

The sustained activity of the animals during the TUNL test sessions (the latencies and number of trials per minute) is a further indicator of the suitability of this procedure for drug testing that requires a low variability in behaviour between animals [23]. Our system could easily be expanded with an automated drug delivery system that dispenses drugs within the sorter. A liquid feeder could deliver a drug and the animal would consume its dose before being admitted to the operant cage [24]. This would synchronise the onset of the drug effect with the individual test phase. Admission to the sorter compartment for drug administration alone would also be possible.

With this fully-automated setup, longitudinal and uninterrupted behavioural testing is in synchrony with the animals’ normal activity rhythm. When animals are transferred by hand to the testing apparatus, their motivation to perform the task cannot be standardized. Moreover, whether an animal is awake or asleep shortly before the test influences the test outcome [25,26].

The fully-automated approach is also advantageous for the experimenter. In the current study, a reduction in daily experimental time of up to 80% was experienced, and the cost of equipment was significantly reduced. In the training phase, one rat is usually given one training session (60 min duration) per day and requires between 10 and 30 sessions before entering the testing phase [13]. Considering the time required to handle one rat before and after each session (i.e., 2 x 10 min = 20 min), a group of six rats using only one operant cage would require a total of 2 h of handling-related time and six hours of training (a total of 8 h per day) for 10 to 30 days (1 session per day). With our system (with only one operant cage available), the time was reduced to a maximum of 2 h, including the weighing of the rats, checking the system, altering schedules and exporting data for analysis. Moreover, the added possibility of training the animals for 24 hours per day drastically reduced the total number of sessions and days required as it was also found in single-housed mice using and automated system for testing cognitive flexibility [27]. One touchscreen system was used for six rats; however, this could be increased to 12 rats with a single touchscreen connected to a second home cage system (Fig 1), with the two groups kept under opposing light/dark cycles. Other types of operant chambers may also be easily attached to the sorter, and the training conditions established here could be adapted to a large variety of tests. However, the extent to which automation will increase reproducibility of results between laboratories remains unknown. Its use in an increased number of laboratories would allow such a comparison.

## Supporting Information

S1 Table (**A)** Details of the protocols described in Oomen et al. (2013), Talpos et al. (2010) and our study. TS: Touchscreen. In Training 4 and Training 6, the criteria required to reach the next phase are more stringent than during the other training phases (2, 3 and 5), with 50 trials/session to be completed during two consecutive sessions **(B)** Detailed list of differences between the protocol described in Talpos et al. (2010) and the current study during the TUNL acquisition task and the TUNL probe test. Adj: adjacent, Med: medium, Max: maximum.
(PDF)

S2 Table Parameters measured during training. *: the formula in Training 6 differs from other training phases; Underlining: indicates a change compared to the previous phase.
(PDF)

S3 Table Comparison of results between the experimental groups of this study. Data from both experimental groups (G1 and G2) were compared for each training phase and for each variable assessed in order to test for consistency and repeatability of the behaviours expressed in this setup (Mann-Whitney U-test for independent samples). The Bonferroni procedure was applied to correct for the multiple testing between the two groups. Following Bonferroni correction, no differences between groups were significant (45 parameters compared in total) except in the Interference test where the % of correct choices (averaged at "large" separation and 2s delay) was higher in G1 than G2 (ITI = 20 sec) but this was not the case with the ITI = 15s. The latency before exiting the operant chamber was the averaged latency per session per animal during the entire training phase. No statistical analysis could be performed regarding animal responses during Training 1 (no individual identification). n.s.: non significant, N.A: non applicable. Further results not included in the table can be found in the main text but were not used for the group comparison.
(PDF)
