# Correction: An Automated, Experimenter-Free Method for the Standardised, Operant Cognitive Testing of Rats

**DOI:** 10.1371/journal.pone.0176807

**Published:** 2017-04-26

**Authors:** Marion Rivalan, Humaira Munawar, Anna Fuchs, York Winter

The Competing Interests statement is incorrect. The correct statement is: YW owns equity in PhenoSys. The other authors have declared that no competing interests exist. This does not alter our adherence to PLOS ONE policies on sharing data and materials.

The following information is missing from the Data Availability statement: Data are available from https://zenodo.org/record/400271.

## References

[1] Rivalan M, Munawar H, Fuchs A, Winter Y (2017) An Automated, Experimenter-Free Method for the Standardised, Operant Cognitive Testing of Rats. PLoS ONE 12(1): e0169476 https://doi.org/10.1371/journal.pone.0169476 28060883 10.1371/journal.pone.0169476 PMC5218494

