# Supplementary material for: An Automated, Experimenter-Free Method for the Standardised, Operant Cognitive Testing of Rats
Source: PLoS One. 2017 Jan 6;12(1):e0169476. doi: 10.1371/journal.pone.0169476 (PMC5218494; doi:10.1371/journal.pone.0169476)
Supplement: S1 Table — (A) Details of the protocols described in Oomen et al. (2013), Talpos et al. (2010) and our study. TS: Touchscreen. In Training 4 and Training 6, the criteria required to reach the next phase are more stringent than during the other training phases (2, 3 and 5), with 50 trials/session to be completed during two consecutive sessions (B) Detailed list of differences between the protocol described in Talpos et al. (2010) and the current study during the TUNL acquisition task and the TUNL probe test. Adj: adjacent, Med: medium, Max: maximum. (PDF) [file pone.0169476.s001.pdf]

An automated, experimenter-free method for the standardised, operant cognitive testing of rats.

Rivalan M*, Munawar H, Fuchs A, Winter Y*.

Department of Biology, Humboldt University of Berlin, Berlin, Germany

* Corresponding authors

E-mails: marion.rivalan@charite.de (MR), york.winter@charite.de (YW)

S1 Table. (A) Details of the protocols described in Oomen et al. (2013), Talpos et al. (2010) and our study. **(B)** Detailed list of differences between the protocol described in Talpos et al. (2010) and the current study during the TUNL acquisition task and the TUNL probe test.

(A)

Oomen et al. 2013		Talpos et al. 2010		Current study	
**Stage 1. Habituation**	**1.** Minimum 2 sessions per rat<br>**2.** pellets added manually in the food tray<br>**3.** Criterion: consuming all rewards in one session<br>**4.** No task related software is active during habituation<br>**5.** Session duration: 30 min	**Habituation**	**1.** Rats manually placed in TS chamber and food pellets were placed manually in the food receptacle and on the shelf<br>**2.** TS not activated but mask was attached to the TS<br>**3.** Session Duration: 20 min	**Training 1 – Habituation/ exploration phase**	**1.** All 15 response windows lit<br>**2.** Image response time 15 min (max) or until nose poke<br>**3.** Condition for reward: no poke within 15 min, all windows turn off and 1 pellet is delivered or one poke on lit window, all windows turn off and 2 pellets are delivered<br>**4.** Sorter deactivated: Gates are open (all rats can enter at once into TS chamber)<br>**5.** Unlimited trials<br>**6.** ITI: 30 sec<br>**7.** Session duration: 24h
**Stage 2. Training to associate stimuli on the screen with a reward**	**1.** All 15 response windows lit<br>Image response time 30 sec or until nose poke<br>**2.** Condition for reward: no response for 30 sec, windows go black 1 pellet delivered. Nose poke on lit window, 3 pellets delivered<br>**3.** Criterion: 60 trials within 60 min<br>**4.** Session duration: 60 min or 100 trials	**Initial touch all lit**	**1.** All response windows lit<br>**2.** Image response time 30sec<br>**3.** Condition for reward: no response for 30 sec, windows go black 1 pellet delivered. Nose poke on lit window, 3 pellets delivered<br>**4.** Criterion: 100 trials in one session (no time cut-off) only 1 session was given per rat	**Training 2 – Initial touch**	**1.** All 15 response windows lit<br>**2.** Image response time 30 sec or until nose poke<br>**3.** Condition for reward: no response for 30 sec, windows go black 1 pellet delivered. Nose poke on lit window, 2 pellets delivered<br>**4.** Criterion to next phase: 17 trials per session in two sessions<br>**5.** Session duration: 10 min or 17 trials<br>**6.** Sorter activated (only one rat at a time is allowed to enter the TS chamber)<br>**7.** 14 Session max in 24h<br>**8.** ITI: 20 sec<br>**9.** Min intersession interval: 30 min
**Stage 3. Training to touch stimuli on the screen for a reward**	**Stage 3 consists in three different phases (i), (ii), (iii):**<br>**(i)** Repeat the procedure as detailed in (stage 2). Start this stage by subjecting animals to this task with all 15 response windows activated simultaneously until criterion is reached.<br>**(ii)** Next, repeat stage 3, but use an adapted version of the task in which a smaller response location is used that consists of four (2 × 2) adjacent locations instead of all 15 windows. The location of this square on the screen should be selected pseudo-randomly across trials.<br>**(iii)** After animals have reached criterion (same as stage 2), repeat stage 3 a third time; use a single window as the active response location. Proceed to stage 4 when the animals have reached criterion (same as stage 2).	**Must touch all lit**	**1.** All response windows lit<br>**2.** Image response time was until nose poke<br>**3.** Condition for reward: nose poke on lit window, 1 pellet delivered<br>**4.** Session duration: 30min or 100 trials<br>**5.** ITI = 5sec<br>**6.** 3 sessions per animal	**Training 3 – Touch all lit windows**	**1.** All 15 response windows lit until nose poke<br>**2.** Image response time was until nose poke<br>**3.** Condition for reward: nose poke on any of the 15 lit windows = 1 pellet delivered<br>**4.** Criterion to next phase: 50 trials per session in two sessions<br>**5.** Session duration: 30 min or 50 trials<br>**6.** Sorter activated (only one rat at time is allowed to enter the TS chamber<br>**7.** 6 sessions max in 24h<br>**8.** ITI: 20 sec<br>**9.** Min intersession interval: 2.5 h
				**Training 4 – Touching one lit window**	**1.** One response window lit (until nose poke) per trial (random location between trials)<br>**2.** Image response time was until nose poke<br>**3.** Condition for reward: nose poke on lit window, 1-pellet delivered<br>**4.** Criterion to next phase: 50 trials per session in two consecutive sessions<br>**5.** Session duration: 30 min or 50 trials<br>**6.** Sorter activated (only one rat at a time is allowed to enter the TS chamber<br>**7.** 6 sessions max in 24h<br>**8.** ITI: 20 sec<br>**9.** Min intersession interval: 2.5 h
**Stage 4. Training to initiate trials**	1. One response window lit per trial (random)<br>**2.** Image response time was until nose poke<br>**3.** Condition for reward: nose poke on lit window, 1-pellet delivered<br>**4.** Criterion: 60 trials within 60 min<br>**5.** Session duration: 60min or 100 trials<br>**6.** Rats received a free pellet at the start of a session so that they initiated the trials	**Must initiate**	**1.** One response window lit per trial (random)<br>**2.** Image response time was until nose poke<br>**3.** Condition for reward: nose poke on lit window, 1-pellet delivered<br>**4.** ITI = 5sec<br>**5.** Session duration: 60min or 100 trials Only 8 response windows were used.<br>**6.** Blank pokes were not punished or rewarded.<br>ITI = 5sec	**Training 5 – Initiating**	**1.** One response window lit per trial (random)<br>**2.** Image response time was until nose poke<br>**3.** Condition for reward: nose poke on lit window, 1-pellet delivered<br>**4.** Criterion to next phase: 50 trials per session in two sessions<br>**5.** Session duration: 30 min or 50 trials<br>**6.** Rats received a free pellet at the start of the session so that they initiated the trials<br>**7.** Sorter activated (only one rat at a time is allowed to enter the TS chamber<br>**8.** 4 Sessions max in 24h<br>**9.** ITI: 20 sec<br>**10.** Min intersession interval: 2.5 h
**Stage 5. Punishment for incorrect responses**	Schedule same as **stage 4 (Training to initiate trials)** but the criterion is ≥ 80% correct within 60 min on two consecutive sessions			**Training 6 – Punishment for incorrect choices**	Schedule same as training 5 (Initiating) but the criterion to next phase is to complete 50 trials with ≥80% correct choices on two consecutive sessions.

Notes S1-A Table: TS: Touchscreen. In Training 4 and Training 6, the criteria required to reach the next phase are more stringent than during the other training phases (2, 3 and 5), with 50 trials/session to be completed during two consecutive sessions

(B)

		ITI (s)	Delay sample to choice phase (s)	Max no. of trials	Size of stimulus (cm)	Separation between stimuli	Start of a session	No. of stimuli on screen
TUNL acquisition task	Current study (Fig. 4)	20	2<br>(recommended by Oomen et al,. 2013)	84	3.3x3.3<br>(as in Oomen et al,. 2013)	Adj (0),<br>Med (1, 2), Max (3)	Automated	3x5<br>(as in Oomen et al,. 2013)
	Talpos 2010 (Fig4)	10	6	64	4.25 x 4.25	Adj (0),<br>Med (1), Max (2)	Manual	3x4<br>(windows of the top row were never lit=8 locations used)
TUNL probe test	Current study (Fig. 5A; Fig. 6)	20	2 and 6	84	3.3x3.3<br>(as in Oomen et al,. 2013)	Adj (0),<br>Med (1, 2), Max (3)	Automated	3x5<br>(as in Oomen et al,. 2013)
	Talpos 2010 (Fig.6)	10	1 and 6	64	2x2	Adj (<3), Med<br>(3 ≥ n <6),<br>Max (≥6)	Manual	3x7<br>(windows of the top row were never lit =14 locations used)

Notes S1-B Table: Adj: adjacent, Med: medium, Max: maximum
