# Supplementary material for: An Automated, Experimenter-Free Method for the Standardised, Operant Cognitive Testing of Rats
Source: PLoS One. 2017 Jan 6;12(1):e0169476. doi: 10.1371/journal.pone.0169476 (PMC5218494; doi:10.1371/journal.pone.0169476)
Supplement: S2 Table — *: the formula in Training 6 differs from other training phases; Underlining: indicates a change compared to the previous phase. (PDF) [file pone.0169476.s002.pdf]

An automated, experimenter-free method for the standardised, operant cognitive testing of rats.

Rivalan M*, Munawar H, Fuchs A, Winter Y*.

Department of Biology, Humboldt University of Berlin, Berlin, Germany

* Corresponding authors

E-mails: marion.rivalan@charite.de (MR), york.winter@charite.de (YW)

S2 Table. Parameters measured during training

**Training phase**	**Parameters measured**
Training 1 – Habituation/exploration phase	**For each animal**<br>• latency to enter the operant chamber for the first time since the beginning of the phase<br>• number of visits of the operant chamber<br>• mean duration of a visit of the operant chamber in total and during the dark or light phases<br>**For the group:** total number of trials, total number of trials with windows touch (%)
Training 2 – Initial touch	**For each animal**<br>• number of entries (or sessions) in the operant chamber<br>• number of detections at each reader (1,2,3) of the sorter<br>• number of trials per session<br>• number of nose pokes on the screen per session<br>• time spent in the chamber in total, and after task completion<br>• percentage of active trials per session
Training 3 – Touch all lit windows	**For each animal**<br>• number of entries (or sessions) in the operant chamber<br>• number of trials per session<br>• number of nose pokes on the screen per session<br>• time spent in the chamber in total, and after task completion<br>• percentage of correct trials per session
Training 4 – Touching one lit window	**For each animal**<br>• number of entries (or sessions) in the operant chamber<br>• number of trials per session<br>• number of nose pokes on the screen per session<br>• time spent in the chamber in total, and after task completion<br>• percentage of correct trials per session
Training 5 – Initiating	**For each animal**<br>• number of entries (or sessions) in the operant chamber<br>• number of trials per session<br>• number of correct and incorrect nose pokes per session (on lit or unlit windows, respectively)<br>• time spent in the chamber in total and after task completion<br>• percentage of correct trials per session
Training 6 – Punishment for incorrect choices	**For each animal**<br>• number of entries (or sessions) in the operant chamber<br>• number of trials per session<br>• number of correct and incorrect nose pokes per session (on lit or unlit windows, respectively)<br>• time spent in the chamber in total and after task completion<br>• percentage of correct* trials per session

Notes S2 Table: *: the formula in training 6 is different from other training phases; Underlining: indicates a change compared to previous phase.
