# Supplementary material for: An Automated, Experimenter-Free Method for the Standardised, Operant Cognitive Testing of Rats
Source: PLoS One. 2017 Jan 6;12(1):e0169476. doi: 10.1371/journal.pone.0169476 (PMC5218494; doi:10.1371/journal.pone.0169476)
Supplement: S3 Table — Data from both experimental groups (G1 and G2) were compared for each training phase and for each variable assessed in order to test for consistency and repeatability of the behaviours expressed in this setup (Mann-Whitney U-test for independent samples). The Bonferroni procedure was applied to correct for the multiple testing between the two groups. Following Bonferroni correction, no differences between groups were significant (45 parameters compared in total) except in the Interference test where the % of correct choices (averaged at "large" separation and 2s delay) was higher in G1 than G2 (ITI = 20 sec) but this was not the case with the ITI = 15s. The latency before exiting the operant chamber was the averaged latency per session per animal during the entire training phase. No statistical analysis could be performed regarding animal responses during Training 1 (no individual identification). n.s.: non significant, N.A: non applicable. Further results not included in the table can be found in the main text but were not used for the group comparison. (PDF) [file pone.0169476.s003.pdf]

An automated, experimenter-free method for the standardised, operant cognitive testing of rats.

Rivalan M*, Munawar H, Fuchs A, Winter Y*.

Department of Biology, Humboldt University of Berlin, Berlin, Germany

* Corresponding authors

E-mails: marion.rivalan@charite.de (MR), york.winter@charite.de (YW)

S3 Table. Comparison of results between the experimental groups of this study.

Variable	Training phase	Measured parameters (per individual)	Statistical test	Outcome
general activity and motivation to enter	Training1	total number of visits in 24h	Mann-Whitney w. bonferroni correction U=min(22.5,13.5)=13.5, n.s.	G1=G2
general activity and motivation to enter	Training1	mean duration of visits during dark (=active) phase	Mann-Whitney w. bonferroni correction U=min(12, 24)=12, n.s.	G1=G2
activity related to reward	Training1	total number of trials in 24h	N.A	G1 (686)< G2 (880)
activity related to reward	Training1	number of trials with windows touch (%) in 24h	N.A	G1 (97.6%) = G2 (98.3%)
motivation to enter	Training1	latency to first entry in the operant chamber	Mann-Whitney w. bonferroni correction U=min(6,30)=6, n.s.	G1=G2
general activity and motivation to enter	Training1	total number of visits during dark (=active) phase	Mann-Whitney w. bonferroni correction U=min(21,15)=15, n.s.	G1=G2
motivation to train	Training2	total number of sessions to reach 80% correct touches	Mann-Whitney w. bonferroni correction U=(11.5,24.5)=11.5, n.s.	G1=G2
motivation to exit	Training2	mean latency to exit the operant chamber after a session is completed	Mann-Whitney w. bonferroni correction U=(22,14)=14, n.s.	G1=G2
motivation to train	Training3	total number of sessions to reach 80% correct touches	Mann-Whitney w. bonferroni correction U=min(6,30)=6, n.s.	G1=G2
motivation to exit	Training3	mean latency to exit the operant chamber after a session is completed	Mann-Whitney w. bonferroni correction U=(19,17)=17, n.s.	G1=G2
motivation to train	Training4	total number of sessions to reach 80% correct touches	Mann-Whitney w. bonferroni correction U=(24, 12)=12, n.s.	G1=G2
motivation to perform	Training4	total number of incorrect touches (last session)	Mann-Whitney w. bonferroni correction U=(17.5,18.5)=17.5, n.s.	G1=G2
motivation to exit	Training4	mean latency to exit the operant chamber after a session is completed	Mann-Whitney w. bonferroni correction U=(31,5)=5, n.s.	G1=G2
motivation to train	Training5	total number of sessions to reach 80% correct touches	Mann-Whitney w. bonferroni correction U=(27,9)=9, n.s.	G1=G2
motivation to perform	Training5	total number of incorrect touches (last session)	Mann-Whitney w. bonferroni correction U=(10.5,25.5)=10.5, n.s.	G1=G2

motivation to exit	Training5	mean latency to exit the operant chamber after a session is completed	Mann-Whitney w. bonferroni correction<br>U=(26,10)=10, n.s.	G1=G2
motivation to train	Training6	total number of sessions to reach 80% correct touches	Mann-Whitney w. bonferroni correction<br>U=(20,16)=16, n.s.	G1=G2
motivation to perform	Training6	total number of incorrect touches (last session)	Mann-Whitney w. bonferroni correction<br>U=(13,23)=13, n.s.	G1=G2
motivation to exit	Training6	mean latency to exit the operant chamber after a session is completed	Mann-Whitney w. bonferroni correction<br>U=(28,8)=8, n.s.	G1=G3
acquisition learning	TUNL	% correct (averaged per blocks)	Mann-Whitney w. bonferroni correction<br>block1: U=(10,26)=10, n.s.<br>block2: U=(8,28)=8, n.s.<br>block3: U=(5,31)=5, 0.025<p<0.05 bonferroni correction p<0.0125<br>block4: U=(8,28)=8, n.s.	G1=G2
acquisition learning	TUNL	total number of correction trials per blocks	Mann-Whitney w. bonferroni correction<br>block1: U=(19,17)=17, n.s<br>block2: U=(9,27)=9, n.s.<br>block3: U=(15,21)=15, n.s.<br>block4: U=(16,20)=16, n.s.	G1=G2
effect of delay between phases and of the distance of the stimulus on performance	Probe test	% correct (averaged per separation distance and delays)	Mann-Whitney w. bonferroni correction<br><u>separation 0</u><br>delay 2sec: U=(13,23)=13, n.s.<br>delay 6sec: U=(23,13)=13, n.s.<br><u>separation 1,2</u><br>delay 2sec: U=(10,26)=10, n.s.<br>delay 6sec: U=(1,35)=1, n.s.<br><u>separation 3</u><br>delay 2sec: U=(4,32)=4, n.s.<br>delay 6sec: U=(11,25)=11, n.s.	G1=G2
motivation to collect reward	Probe test	mean latency to collect reward	Mann-Whitney w. bonferroni correction<br><u>separation 0</u><br>delay 2sec: U=(3,33)=13, n.s.<br>delay 6sec: U=(22,14)=14, n.s.<br><u>separation 1,2</u><br>delay 2sec: U=(0,36)=0, n.s.<br>delay 6sec: U=(0,36)=0, n.s.<br><u>separation 3</u><br>delay 2sec: U=(6,36)=6, n.s.<br>delay 6sec: U=(16, 20)=16, n.s.	G1=G2
motivation to make a choice	Probe test	mean latency to "correct Image Response" (choice phase only)	Mann-Whitney w. bonferroni correction<br><u>separation 0</u><br>delay 2sec: U=(22,14)=14, n.s.<br>delay 6sec: U=(23,13)=13, n.s.<br><u>separation 1,2</u><br>delay 2sec: U=(23,13)=13, n.s.<br>delay 6sec: U=(25,11)=11, n.s.<br><u>separation 3</u><br>delay 2sec: U=(31,5)=5, n.s.<br>delay 6sec: U=(27,9)=9, n.s.	G1=G2

effect of previous trial on next trial	Interference test	%correct (averaged at "large" separation and 2s delay but different ITI)	Mann-Whitney w. bonferroni correction separation 3, delay 2 sec:<br>ITI 20 sec: U=(4,32)=45, p<0.025<br>ITI 15 sec: U=(7,29)=7, n.s.	G1>G2 (ITI20)<br>G1=G2 (ITI15)

Notes S3 Table: Data from both experimental groups (G1 and G2) were compared for each training phase and for each variable assessed in order to test for consistency and repeatability of the behaviours expressed in this setup (Mann-Whitney U-test for independent samples). The Bonferroni procedure was applied to correct for the multiple testing between the two groups. Following Bonferroni correction, no differences between groups were significant (45 parameters compared in total) except in the Interference test where the % of correct choices (averaged at "large" separation and 2s delay) was higher in G1 than G2 (ITI=20 sec) but this was not the case with the ITI=15s. The latency before exiting the operant chamber was the averaged latency per session per animal during the entire training phase. No statistical analysis could be performed regarding animal responses during Training 1 (no individual identification). n.s.: non significant, N.A: non applicable. Further results not included in the table can be found in the main text but were not used for the group comparison.
